# Seed priming with plant waste extracts enhances maize drought tolerance in a genotype-specific manner

**DOI:** 10.3389/fpls.2025.1717255

**Published:** 2025-11-10

**Authors:** Hisham Wazeer, Ahmad Zeidan, Jacopo Allevi, Andrea Pagano, Conrado Dueñas, Adriano Marocco, Lorenzo Stagnati, Enrico Doria, Anca Macovei

**Affiliations:** 1 Department of Biology and Biotechnology ‘L. Spallanzani’, University of Pavia, Pavia, Italy; 2 Department of Sustainable Crop Production, Università Cattolica del Sacro Cuore, Piacenza, Italy; 3 Research Centre for Biodiversity and Ancient DNA, Università Cattolica del Sacro Cuore, Piacenza, Italy

**Keywords:** biostimulants, drought, seed germination, sustainable agriculture, *Zea mays*

## Abstract

Climate change poses major challenges to the agri-food sector, with drought episodes, defined as extended periods of limited water availability, representing one of the most damaging stress factors. While drought tolerance has been extensively studied during vegetative and reproductive stages, its effects on seed germination remain comparatively underexplored. This is particularly relevant for maize (*Zea mays*), a globally relevant staple crop whose productivity is highly sensitive to water deficit, making the identification of drought-tolerant varieties a high priority. Addressing this challenge requires sustainable agricultural practices. Plant-based biostimulants (PBs), derived from natural sources, are gaining attention for their ability to promote plant growth, enhance stress resilience, and reduce reliance on synthetic fertilizers, thus supporting both bioeconomy and environmental sustainability. Similarly, seed priming, a pre-sowing technique that improves germination efficiency, offers an additional strategy to strengthen crop resilience. In this study, 26 Italian maize genotypes were evaluated for their drought-tolerance potential. Biostimulants produced from plant waste by-products (red chicory and cauliflower extracts) were applied as seed priming agents and the germination performance under drought stress was monitored over a 14 days period. The germination behavior was evaluated by calculating several parameters, including germination percentage, speed, seedling growth, and stress tolerance index. In addition, reactive oxygen species (ROS) accumulation was quantified in the dry seeds of selected genotypes, alongside the expression of genes involved in ROS homeostasis and drought response. The obtained results reveal genotype-dependent responses to drought and demonstrate that priming treatments enhanced both drought tolerance and germination performance in several maize genotypes. This study emphasizes the value of genetic diversity and highlights the potential of seed priming with plant-based biostimulants as a sustainable strategy within the framework of circular bioeconomy and climate-resilient agriculture.

## 1 Introduction

As climate change progresses, there is growing evidence of a global rise in both the frequency and intensity of drought events (Savelli et al., 2022). The interplay between rising temperatures and drought conditions significantly affects ecosystem dynamics, as elevated heat can amplify drought effects, increase plant stress and gradually transform ecosystem structure and composition (van der Sande et al., 2023). This phenomenon adversely affects agricultural output particularly in vulnerable regions where food security is already compromised (Myers et al., 2017).

Maize (*Zea mays* L.) is a cornerstone of the global economy, providing substantial economic value as a versatile crop for human consumption, animal feed, and industrial processing (Palacios-Rojas et al., 2020). While its C_4_ photosynthetic pathway provides advantages in carbon dioxide utilization and heat tolerance, maize remains vulnerable to extreme climatic conditions, which can damage reproductive organs and reduce yield (Guidi et al., 2019). The rising temperatures are increasingly compromising the plant’s innate defense mechanisms, as heat stress can disrupt the biosynthesis and regulation of defensive metabolites, making maize more susceptible to pests and diseases (Yactayo-Chang and Block, 2023). Crop breeding programs have met significant milestones not only in enhancing yield but also in improving traits like drought tolerance, heat resistance, and disease defense. New maize cultivars with refined root systems and stress-responsive genes are being developed to better withstand both abiotic and biotic stressors (Feng et al., 2022; Li et al., 2023; Li et al., 2024; Chavhan et al., 2025). Recent advances in biotechnology, particularly the application of CRISPR/Cas9, are transforming maize improvement by enabling accurate modification of drought-responsive genes (Namata et al., 2025). For instance, editing of *GA20ox3* (Gibberellin 20-oxidase 3) has been shown to improve drought tolerance while maintaining yield by altering gibberellin biosynthesis and stress-related hormone accumulation (Liu et al., 2023). Similarly, targeted knockout of *ZmPL1* (Phylloplanin-like) resulted in improved seedling survival, reduced oxidative damage, and stronger antioxidant responses under water scarcity (Wang et al., 2025).

Despite these advancements, research on maize seed germination in the drought context remains limited. Germination marks the initial phase of plant development, beginning with water uptake and ending in radicle emergence. This process is fundamental not only for successful seedling establishment, but also in the context of the final crop yield (Han and Yang, 2015). Germination progresses through three distinct phases: phase I involves rapid water absorption and seed hydration; phase II encompasses reserve mobilization and the synthesis of new RNA and proteins; and phase III is characterized by radicle protrusion, signifying the transition to post-germination and the loss of desiccation tolerance (Smolikova and Medvedev, 2022). Seed priming, a pre-sowing treatment that involves a partial hydration to initiate early metabolic activities associated with germination, follows these phases but it has to be stopped before entry in phase III (Corbineau et al., 2023; Pagano et al., 2023). This carefully controlled hydration triggers reversible activation of key cellular processes, including DNA repair, enzyme activity, and the antioxidant defense system (Kiran et al., 2020; Pagano et al., 2023; MacDonald and Mohan, 2025). Delayed dry-back can result in “over-priming”, a state in which seeds lose desiccation tolerance, ultimately compromising viability and preventing successful seedling establishment (Pagano et al., 2022).

Seed priming has been efficiently used to improve maize germination and resilience under stress conditions. Different priming agents have been used in recent times to ameliorate the early growth by activating protective mechanisms against abiotic stresses. Silicon-based priming enhanced maize performance under drought stress by regulating morpho-physiological traits and boosting antioxidant metabolism (Parveen et al., 2019). Similarly, melatonin-based priming enhanced drought tolerance in seedlings through a boost in the antioxidant defense and photosynthetic efficiency (Muhammad et al., 2023). Bio-priming with beneficial microorganisms elevated maize tolerance to salt stress by modulating antioxidant activity and regulating stress-responsive miRNA expression (Aydinoglu et al., 2023). Osmopriming with polyethylene glycol (PEG) has been shown to enhance both physiological responses and agronomic attributes under simulated water deficit conditions (Kakar et al., 2023).

Seed priming can be further upgraded by its combination with plant-derived biostimulants (PBs), thus better aligning with the 3R (Reduce, Reuse, Recycle) circular bioeconomy principles. Unlike fertilizers or pesticides, biostimulants enhance plant growth by activating natural physiological processes that improve nutrient efficiency, stress tolerance, and overall vigor (Yakhin et al., 2017). PBs are known to be rich in bioactive compounds and can be derived from various plant materials and agricultural by-products, making them a valuable, eco-friendly solution to transform plant waste into growth-promoting products (Puglia et al., 2021; Mashamaite et al., 2022; Gervasi and Mandalari, 2024; Wazeer et al., 2024). The use of PBs have shown notable positive effects on maize growth and development. Water-extractable organic matter from composted biomasses resulted in enhanced germination and early seedling growth by stimulating root elongation and improving nutrient uptake (Monda et al., 2017). Similarly, extracts from hydrochar derived from sugarcane industry by-products promoted germination, likely due to the presence of bioactive compounds which activate key metabolic pathways (Bento et al., 2021). Lignin nanoparticles, considered as novel PB agents, have improved maize physiological and biochemical traits, including chlorophyll content and antioxidant activity, leading to enhanced stress resilience and plant vigor (Del Buono et al., 2021).

The current study focused on investigating the effects of drought stress during germination in a collection of Italian maize genotypes, enabling their classification as either drought-susceptible or drought-tolerant. Subsequently, seed priming was applied to mitigate the adverse impact of drought on germination. These treatments were based on using plant-derived extracts obtained from agricultural by-products of red chicory (*Cichorium intybus* var. *foliosum*) and cauliflower (*Brassica oleracea* var. *botrytis*). The extracts were obtained using a sustainable extraction procedure (Doria et al., 2022) and prepared as aqueous solutions. For this reason, their effects were compared with both hydropriming (imbibition in water) and untreated controls. Germination responses were evaluated at the phenotypic level in terms of germination percentage, speed, and stress tolerance. A targeted molecular profiling, based on measuring ROS and gene expression profiles, was performed on the dry seeds of selected varieties to evaluate potential seed quality markers.

## 2 Materials and methods

### 2.1 Experimental design

Maize seeds belonging to 26 Italian varieties representing different genetic and agronomic backgrounds (**Supplementary Figure 1**; **Supplementary Table 1**) were used in this study. The seeds are part of a local germplasm collection held at Università Cattolica del Sacro Cuore, Piacenza, Italy.

**Figure 1:**
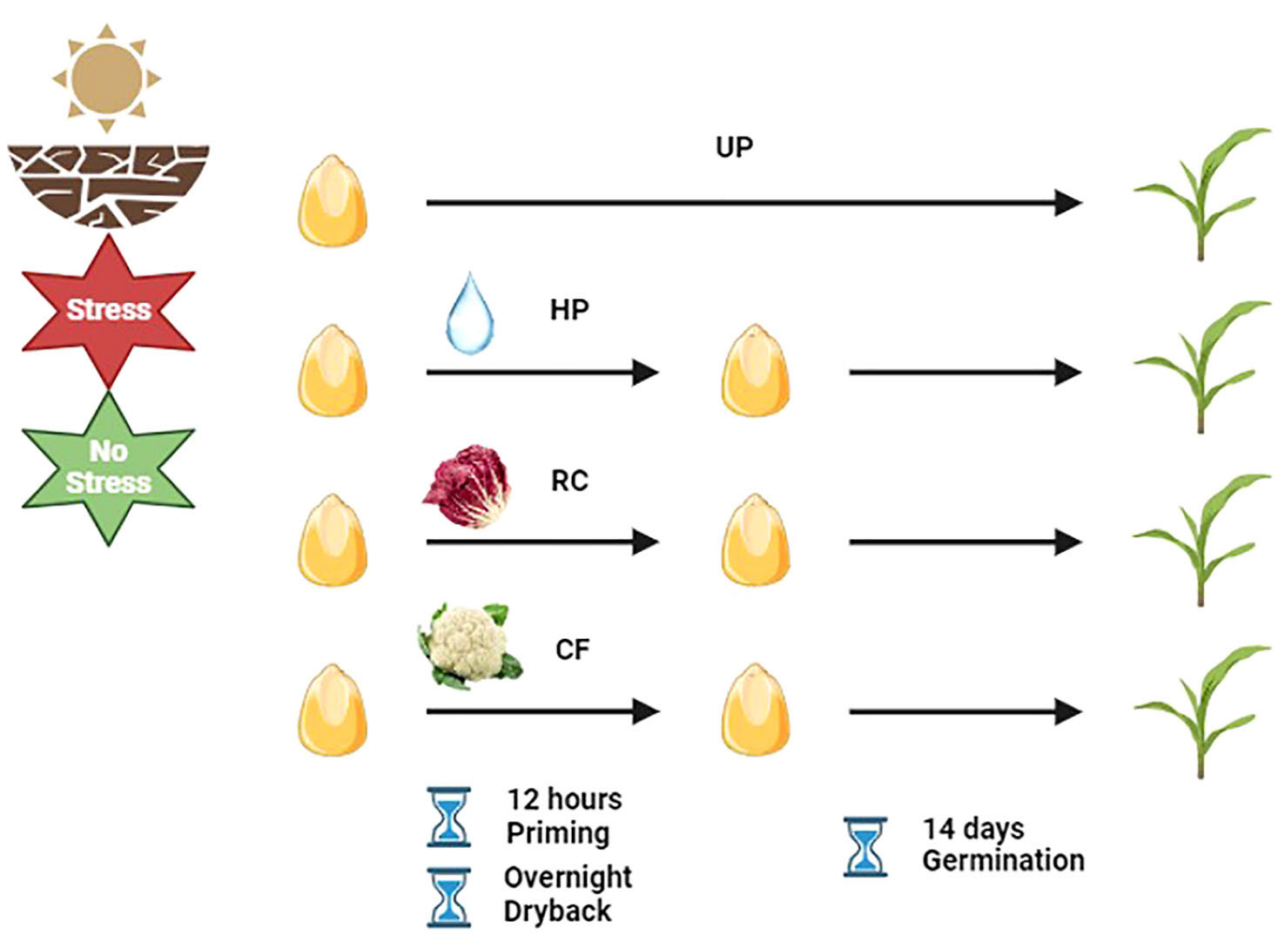
Schematic representation of the experimental system (https://www.biorender.com/). UP, unprimed; HP, hydropriming; RC, priming with red chicory extract; CF, priming with cauliflower extract.

The proposed experimental system (**Figure 1**) combined seed priming with plant-waste extracts and drought treatments applied in a soil system (Dueñas et al., 2024). This was based on using the soil volumetric moisture content (VMC) and designed to simulate two field conditions corresponding to saturated soil (>50% VMC) for optimal growth conditions, and 30% VMC for drought stress. Maize seeds were sown in 8 cm by 11 cm germination trays containing 40 g of oven-dried sieved clay loam soil (VIGORPLANT ITALIA S.r.l, Piacenza, Italy). The amount of added water was determined according to the soil VMC and bulk density (Dueñas et al., 2024). Water addition was carried out on a daily basis to maintain the VMC at the indicated levels. Three seed priming treatments, namely, hydropriming (HP), and priming with red chicory (RC), or cauliflower (CF) extract, were applied while untreated seeds (UP, unprimed) were used as control. For the priming treatments, maize seeds were imbibed in water or the respective plant extract solutions for 12 h and subsequently left to dry overnight at 20°C. Primed/unprimed seeds were germinated in the absence/presence of drought stress. For each treatment combination, 20 seeds were placed in germination trays and three replicates were used for each condition. The trays were kept in a growth chamber at 25°C under light conditions with a photon flux density of 35 μmol m^-2^s^-1^, 19-26% relative humidity, and photoperiod of 16/8 h. The trays were monitored for 14 days.

### 2.2 Plant extracts preparation

Red chicory (*Cichorium intybus* var. *foliosum*) and cauliflower (*Brassica oleracea* var. *botrytis*) waste by-products were weighted, aliquoted, and stored at -45°C (monitored by a datalogger, T184 T4, Liebherr) for over three months. An aliquot of 4.5 kg was thawed at room temperature for 2 days. After draining the residual water, each aliquot was grounded using an industrial mill (M100, Enoitalias.r.l., Florence, Italy). The materials were shattered into small pieces (0.5–1.0 cm in diameter) for subsequent extraction processing. The amount of cut leaves was incubated at 50°C for 4 h with 7 L of water and a commercial enzymatic mixture (Novozyme) described by the following codes: 118, 086, 012, 030. Each mixture was added with a concentration of cca. 0.5 g kg^-1^ of material. The pH was set to 5.0 using phosphoric acid. An isothermal cyclically pressurized extraction process (rapid solid–liquid dynamic extraction) was applied to pretreated material using the Naviglio mechanical extractor (Nuova Estrazione, Naples, Italy). The extraction was performed using only water and was carried on for 5 h at room temperature. The extract was partially concentrated using a rotary evaporator (Rotavapor System R-300, BüchiLabortechnik AG, Flawil, Switzerland) and filtrated before biochemical analysis.

### 2.3 Total polyphenol content and HPLC analyses

The total content of polyphenols was measured using the Total Polyphenols Colorimetric Assay Kit (Steroglass, Perugia, Italy) according to the manufacturer’s instructions. Absorbance was measured at 725 nm and results were expressed in gallic acid equivalents using a gallic acid standard curve.

A 20 µL aliquot of the filtered sample was injected into the HPLC pump (Kontron420; Kontron Instruments, Munich, Germany) equipped with a C18 column (ZORBAX ODS 250 nm 4.6 mm column, 5 um particle size, Sepachrom, Milan, Italy). HPLC analyses were performed at 0.8 mL min-1 flow rate, setting the detector at 280 nm; the mobile phases consisted in 5% acetic acid (A) and pure methanol (B), according to the gradient conditions summarized in **Supplementary Table 2**.

### 2.4 Germination monitoring

Multiple germination parameters were calculated (Dueñas et al., 2024) and the respective equations are illustrated in **Supplementary Table 3**. Given the setup of the experiment, germinated seeds were scored when seedlings emerged from the soil.

The monitored parameters include: G% (germination percentage), the daily percentage of germinated seeds; MGT (mean germination time), the standard time of germination; and GSTI (germination stress tolerance index), the mean ratio between germination under optimal and drought stress conditions. Shoot (SL) and root (RL) length were measured at day 14 using the ImageJ (https://imagej.net/ij/) free online software. To this purpose, six seedlings/treatment were photographed and used to determine the length.

### 2.5 FOX-1 assay

Peroxyl radicals and hydrogen peroxide (H_2_O_2_) concentrations [ROOH] were quantified in control and RC-primed seeds. The assay was carried out by using the xylenol orange reagent (Carlo Erba, Milan, Italy), which reacts with Fe^3+^ (derived from the oxidation of Fe^2+^ induced by peroxyl radicals and H_2_O_2_) to give a blue-violet complex with an absorption maximum at 560 nm (Bridi et al., 2015). The working solution (FOX-1) was prepared as previously described (Griffo et al., 2023). A solution containing ammonium ferrous (II) sulphate (NH_4_)_2_Fe(SO_4_)_2_·6H_2_O 25 mM (Merk’s Reagents, Darmstadt, Germany) in H_2_SO_4_ 0.25 M (Honeywell, Charlotte, NC, USA) was added to a Milli-Q water solution containing xylenol orange 125 μM and D-sorbitol 100 mM (Duchefa Biochemie, Haarlem, The Netherlands) in a ratio of 1:100. The solutions were mixed gently until the color became uniform. Seed samples were incubated in 10 ml of Milli-Q water and 200 μL were transferred to 1 ml of FOX-1 solution. Three replicates of seven seeds each were used per sample. Absorbance was determined at 560 nm by using a Biochrom WPA Biowave (Biochrom Ltd.) spectrophotometer. A calibration curve was performed by using FOX-1 solution with different concentrations (0, 0.25, 0.5, 1.25, 2.5 μM) of H_2_O_2_ for normalization.

### 2.6 RNA extraction, cDNA synthesis and qRT-PCR

Total RNA was isolated from dry seeds of selected genotypes by using TRIzol™ (Thermo Fisher Scientific, Monza, Italia), as indicated by the provider, followed by a DNase (Thermo Fisher Scientific) treatment. RNA was quantified by using NanoDrop (Biowave DNA, WPA, Thermo Fisher Scientific). Subsequently, cDNAs were obtained by using the RevertAid First Strand cDNA Synthesis Kit (Thermo Fisher Scientific) according to the manufacturer’s suggestions. The *q*RT-PCR reactions were performed with the Maxima SYBR Green *q*PCR Master Mix (Thermo Fisher Scientific) according to the supplier’s indications, by using a CFX Duet, Real-Time PCR System (BIO-RAD, Milano, Italy). Amplification conditions were as follows: denaturation at 95°C for 10 min, and 45 cycles of 95°C for 15 s and 60°C for 60 s. Oligonucleotide sequences (**Supplementary Table 4**) were designed by using Primer-BLAST tool of NCBI (https://www.ncbi.nlm.nih.gov/tools/primer-blast/) and verified with Oligo Analyzer Tool (https://eu.idtdna.com/pages/tools/oligoanalyzer) free online tools. Relative quantification was carried out with 18s ribosomal RNA (*18s*, NCBI accession LOC118472325) as reference gene (Lin et al., 2014). The investigated genes were: *MSD3.4* (Mn-superoxide dismutase 3.4; NCBI accession: Zm00001d009990), *CAT1* (catalase 1; NCBI accession: Zm00001d014848), *APX1.1* (ascorbate peroxidase 1.1; NCBI accession: Zm00001d028709), *GST1* (glutathione S-transferase 1; NCBI accession: Zm00001d012675), *TRPP1* (trehalose-6-phosphate phosphatase 1; NCBI accession: Zm00001d032298), *DREB2A* (dehydration-responsive element-binding 2A; NCBI accession: Zm00001d008665), *PMP3g* (plasma membrane proteolipid 3g; NCBI accession: Zm00001d024778), and *LEA1* (Late embryogenesis abundant 1; NCBI accession: Zm00001d027740). Raw fluorescence data provided by Software 1.7 (BIO-RAD) were used to determine the threshold cycle number (Ct) values for each transcript quantification. Relative quantification of transcript accumulation was performed as described by Thomsen et al. (2010) using the X_0_ method in which a conversion of the exponentially related Ct values is introduced to obtain linearly related values, representing the amount of starting material in a qPCR. All reactions were performed in triplicates.

### 2.7 Statistical analyses

Germination parameters data were analyzed with Student *t*-test, using the Microsoft Excel package, and setting the threshold at *p*-value ≤ 0.05, represented with an asterisk (*). Additionally, a multi-factorial ANOVA was conducted in an R background (https://www.r-project.org/) to evaluate the impact of genotype, priming, and stress. The FOX-1 and *q*RT-PCR data were analyzed using the Tukey’s test with the software developed by Assaad et al. (2014), based on the one-way ANOVA and a threshold of *p* ≤ 0.05. Principal Component Analysis (PCA) was carried out with the RStudio (Posit version 2025.05.1 + 513) using the *FactoMineR* package to identify major patterns of variation and reduce data dimensionality. Visualization of the PCA biplots was obtained with the *ggfortify* package, which facilitated the interpretation of sample groupings and variable contributions.

## 3 Results

### 3.1 Characterization of the plant-waste extracts and defining the experimental conditions

A basal characterization of the RC and CF extracts in terms of polyphenol content was performed through HPLC. The obtained data show that the RC extract is rich in quercetin (81.232 µg g^-1^), epicatechin 3 gallate (51.002 µg g^-1^), cinnamic acid (50.211 µg g^-1^), and chlorogenic acid (20.531 µg g^-1^) (**Supplementary Figure 2a**). Similarly, the CF extract is characterized by the presence of chlorogenic acid (51.661 µg g^-1^), rutin (14.607 µg g^-1^), and smaller amounts of other compounds like quercetin (7.971 µg g^-1^), epicatechin (7.176 µg g^-1^), epicatechin 3 gallate (6.566 µg g^-1^), gly-vitexin (5.02 µg g^-1^), cinnamic acid (2.455 µg g^-1^), and cumaric acid (2.106 µg g^-1^) (**Supplementary Figure 2b**). Overall, the RC extract was richer in total polyphenols (2.201 mg g^-1^) compared to the CF extract (1.988 mg g^-1^).

To develop a suitable experimental setup for seed priming, several dilutions of the plant extracts were tested while the selection of the imbibition and dry back timing was based on previous work (Colombo et al., 2023). The preliminary tests consisted in using three dilutions of the CF (x10, x5 and x3) extract applied to a commercial maize variety in the presence/absence of 18% polyethylene glycol (PEG) to simulate water deprivation conditions (**Supplementary Figure 3**). All dilutions used resulted in improved G% at day 2; however, the x5 dilution showed the highest G% under stress conditions from day 3 to 7 (**Supplementary Figures 3c, d**). Regarding the RC extract, previous experiments on multiple species pinpointed the RC x6 dilution as most efficient (data not shown), and was further confirmed in our preliminary testing (**Supplementary Figures 3a, b**). Therefore, the RC x6 and CF x5 dilutions were used for the subsequent experiments.

To select the drought stress based on soil VMC, three different levels were tested, specifically 36%, 30% and 25%, along with the saturated conditions serving as control (**Supplementary Figure 3e**). The selection criterion was based on G% showing a clear reduction without complete inhibition, ensuring partial germination under suboptimal conditions. The obtained results indicated that the 30% VMC condition was most suitable for this purpose, so this treatments was chosen for the subsequent analyses.

### 3.2 Identification of drought tolerant genotypes

**Figure 2:**
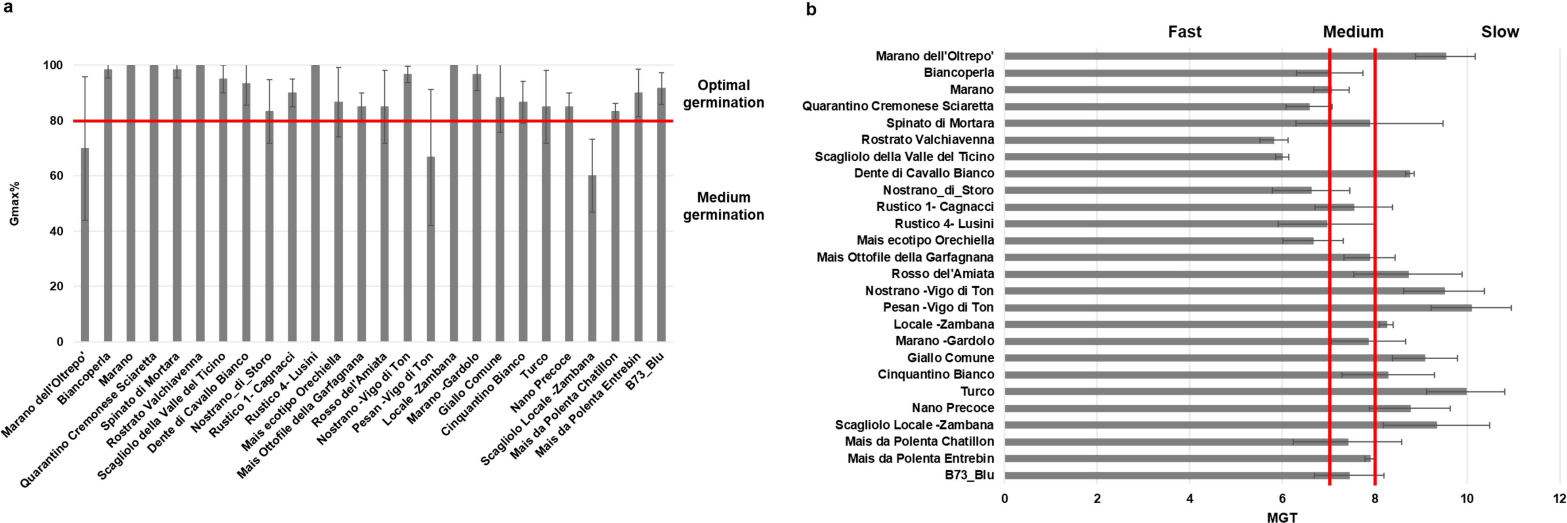
Germination performance of 26 Italian maize varieties under optimal growth conditions. **(a)** Germination percentage (G%). **(b)** Mean germination time (MGT). Data is presented as means ± standard deviations of three independent replicates.

**Figure 3:**
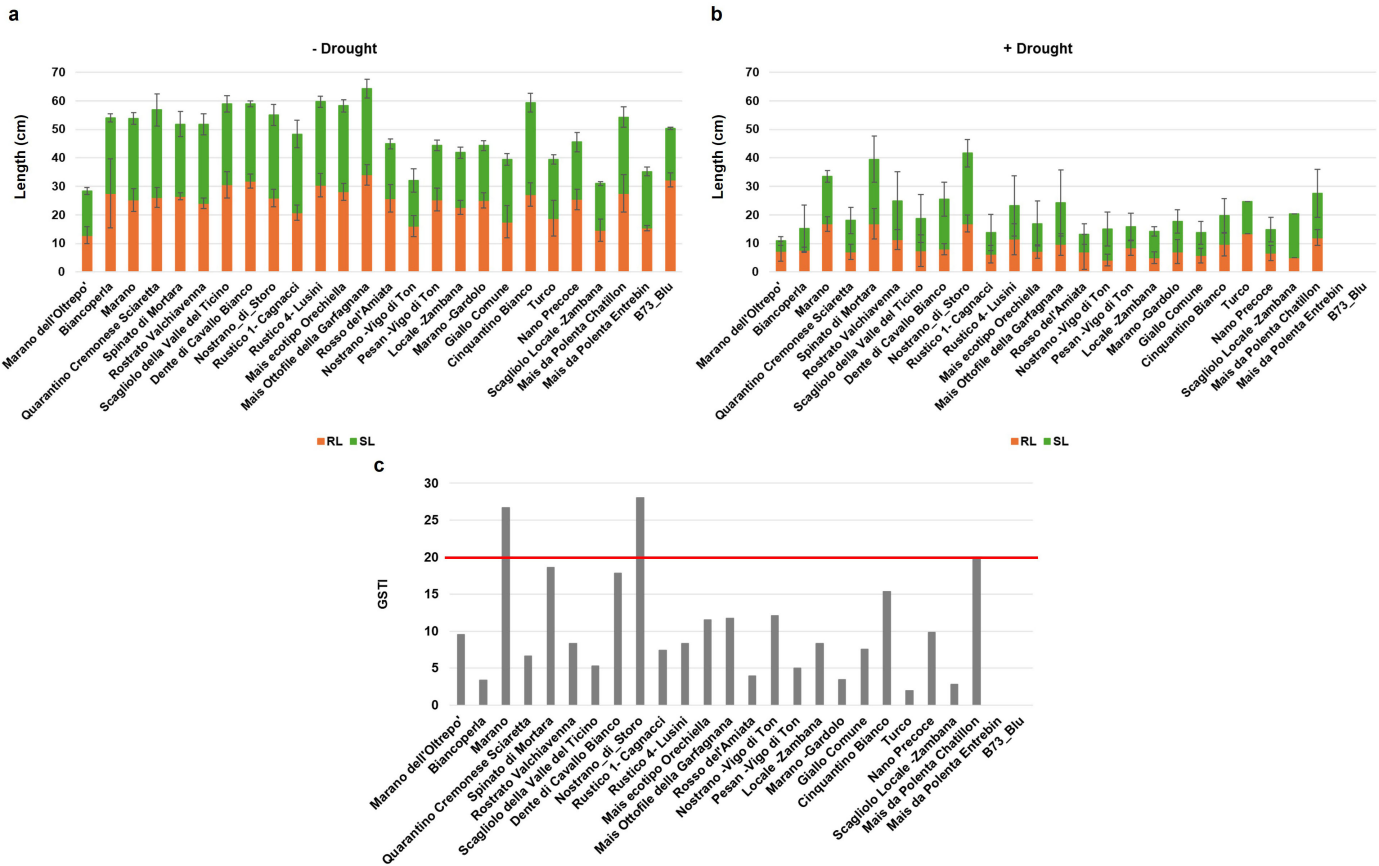
Seedling length of the 26 Italian maize varieties grown for 14 days in the soil. **(a)** Root length (RL) and shoot length (SL) under optimal conditions. **(b)** RL and SL under drought stress. Data is presented as means ± standard deviations of three independent replicates. **(c)** Germination stress tolerance index (GSTI). Data is presented as a ratio of means between the levels of germination under optimal and stress conditions.

The 26 maize genotypes were sown in soil-based germination trays and monitored for a period of 14 days while germination data were collected on daily basis. Based on the G% in the absence of stress, the materials were initially classified in two classes: optimal germination (above 80%) for most varieties, and medium germination (below 80%) observed for Marano dell’Oltrepo, Pesan-Vigo di Ton, and Scagliolo Locale-Zambana genotypes (**Figure 2a**). When considering germination speed in terms of MGT values, the genotypes were further classified as fast, medium or slow germinating (**Figure 2b**); namely, 6 genotypes presented a fast germination (< 7 days), 9 genotypes medium speed (between 7 and 8 days), and 11 genotypes showed a slow germination (> 8 days). The root and shoot length presented high variability among genotypes, where Marano dell’Oltrepo showed the lowest values while Mais ottofile della Garfagnana the highest (**Figure 3a**). When drought stress was applied, seedling growth was substantially decreased (**Figure 3b****).** To better evidence tolerance/sustainability traits, the germination stress tolerance index (GSTI) was calculated. A threshold of 20% was established, where genotypes exhibiting a GSTI ≥ 20% were classified as drought-tolerant, while those with lower values were considered susceptible (**Figure 3c**). Only three genotypes appeared to show drought tolerance at germination, namely Nostrano di Storo (GSTI 28%), Marano (GSTI 26.67%), and Mais da Polenta Chatillon (GSTI 20%).

**Figure 4:**
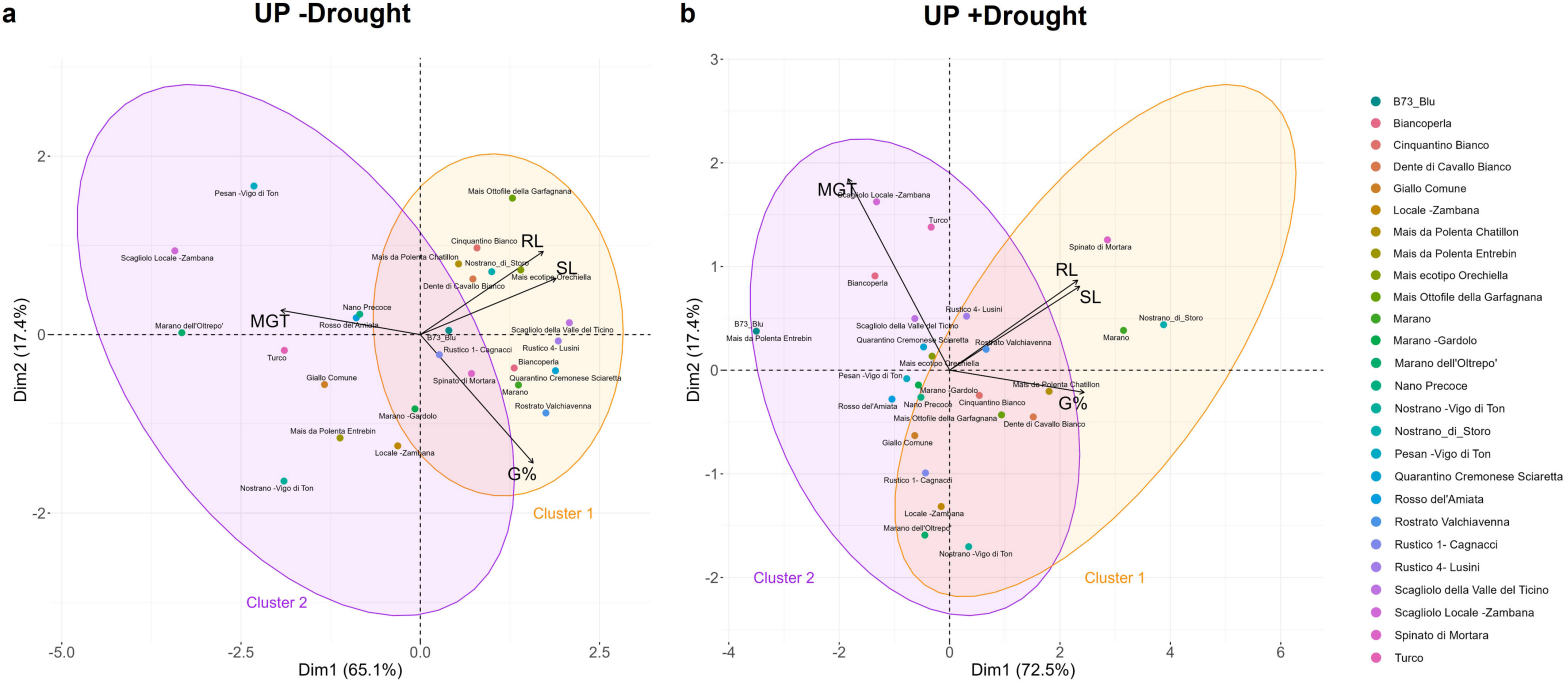
Principal component analysis (PCA) based on all calculated germination parameters. **(a)** PCA plot for germination in the absence of stress (-Drought). **(b)** PCA plots for germination in the presence of stress (+Drought). UP, unprimed; G%, germination percentage; MGT, mean germination time; SL, shoot length; RL, root length.

The PCA analysis conducted using all germination parameters calculated in the presence/absence of drought allowed to differentiate the genotypes in two clusters. In the absence of stress (**Figure 4a**), the PCA score plot, explaining 82.5% (65.1% for Dim1 and 17.4% for Dim2) of the variance between parameters, point at two clusters separated based on optimal (cluster 1 in orange) or medium (cluster 2 in purple) germination classes. Similarly, the two classes obtained in the presence of drought (**Figure 4b**), distinguishes the genotypes based on their tolerance (cluster 1) or susceptibility (cluster 2) to the stress during germination. The contribution of each of the germination variables to this distinction, is reported in the supplementary data (**Supplementary Table 5**).

Overall, the combination of parameters and analyses used, allowed to identify four local maize varieties (Nostrano di Storo, Marano, Mais da Polenta Chatillon, Spinato di Mortara) with a relative drought tolerance capacity at the germination and early seedling stages.

### 3.3 Seed priming with plant extracts can improve drought stress tolerance at germination

**Figure 5:**
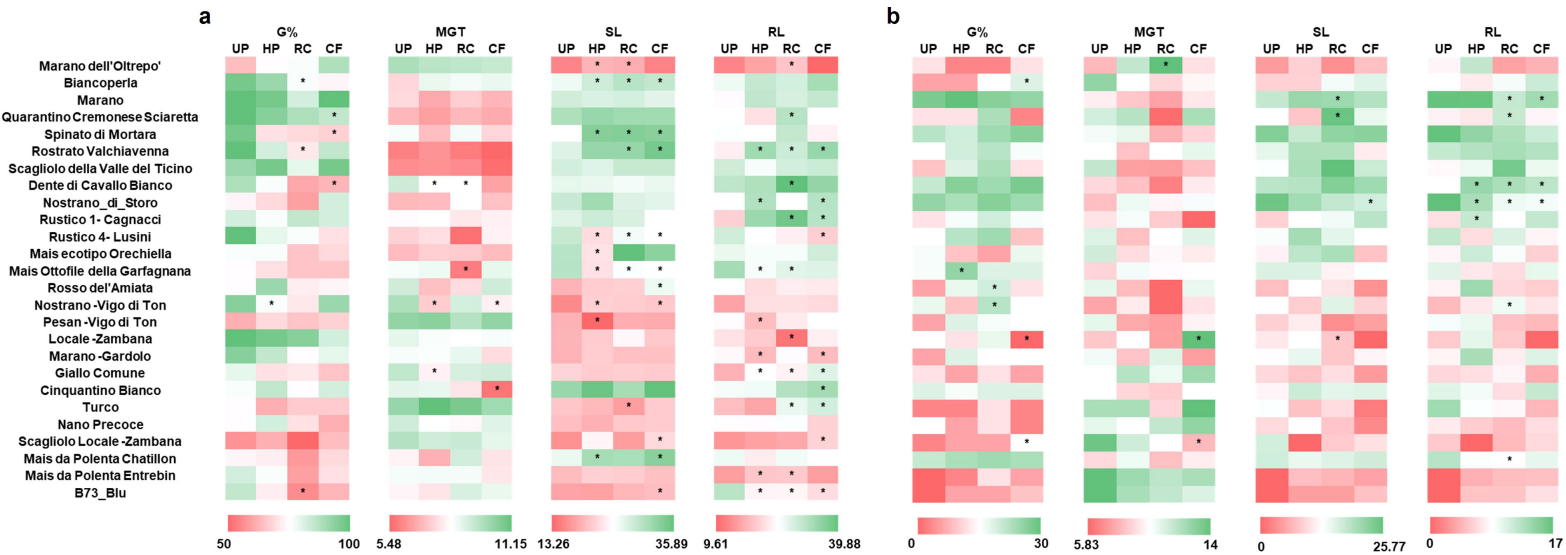
Effect of seed priming on the germination parameters of the 26 Italian maize verities investigated. **(a)** Germination parameters in the absence of stress. **(b)** Germination parameters in the presence of drought stress. Data is presented as a heatmap based on the means values obtained from three independent replicates. Asterisks indicate statistically significant differences (*p ≤ 0.05) to each respective UP control. G%, germination percentage; MGT, mean germination time; SL, shoot length; RL, root length; UP, unprimed; HP, hydropriming; RC, priming with red chicory extract; CF, priming with cauliflower extract.

Given that the majority of the varieties tested resulted susceptible to drought, the next step consisted in identifying priming protocols that can sustainably and significantly mitigate its negative effects during germination. To this purpose, the RC and CF extracts were separately used as priming agents while HP was added for comparison purposes. This is both because the extracts were prepared and diluted in water, as well as the fact that HP is the most common and user-friendly priming method, easily applicable on large scale, field conditions (Harris, 2006). All recorded germination parameters (G%, MGT, SL, RL) were gathered in a heatmap (**Figure 5**) to effectively visualize the large volume of collected data.

Under optimal conditions (**Figure 5a**), a negative impact on G% was evidenced in some cases (e.g., Nostrano -Vigo di Ton with HP; Biancoperla, Rostrato Valchiavenna and B73_blu with RC; Quarantino Cremonese Sciaretta, Spinato di Mortara and Dente di Cavallo Bianco with CF). The MGT values indicated a faster germination in some genotypes depending on the priming methods used (e.g., Dente di Cavallo Bianco, Nostrano -Vigo di Ton and Giallo Comune with HP; Dente di Cavallo Bianco and Mais Ottofile della Garfagnana with RC; Nostrano -Vigo di Ton and Cinquantino Bianco with CF). As for the SL and RL values, many genotypes showed different degrees of statistically significant differences in terms of both positive (e.g., Rostrato Valchiavenna improved root growth in response to all priming methodologies applied) or negative (e.g., B73_Blu diminished root growth in response to all priming methodologies applied) outcomes. These findings support a variety- as well as priming method-dependent response under optimal soil moisture conditions.

Under drought conditions (**Figure 5b**), the G% was significantly improved in four varieties in response to the different priming approaches (Mais Ottofile della Garfagnana with HP; Rosso del’Amiata and Nostrano -Vigo di Ton with RC; Biancoperla with CF). Anticipated germination, in terms of lower MGT values were registered only for one variety (Scagliolo Locale -Zambana) treated with CF. In terms of improved seedling growth (SL/RL), few varieties (e.g., Marano, Quarantino Cremonese Sciaretta, Dente di Cavallo Bianco, Nostrano di Storo) presented this characteristic. The presented data shows that the different priming treatments can have a distinct impact on mitigating drought stress, still in a genotype-dependent manner.

To further discriminate between the impact of genotype, priming and stress, a multifactorial ANOVA was conducted (**Supplementary Table 6**). The data evidenced that each single factor (genotype, treatment, condition) has a significant impact of G%, MGT, SL and RL data. When looking at the interaction between factors, only SL and RL data presented significant values for all comparison levels, namely genotype: treatment, genotype: condition, treatment: condition, genotype: treatment: condition. For the G% and MGT data, significance was obtained only for genotype: condition, and treatment: condition. This may be due to the distribution of the samples, residual normality, or variance homogeneity.

**Figure 6:**
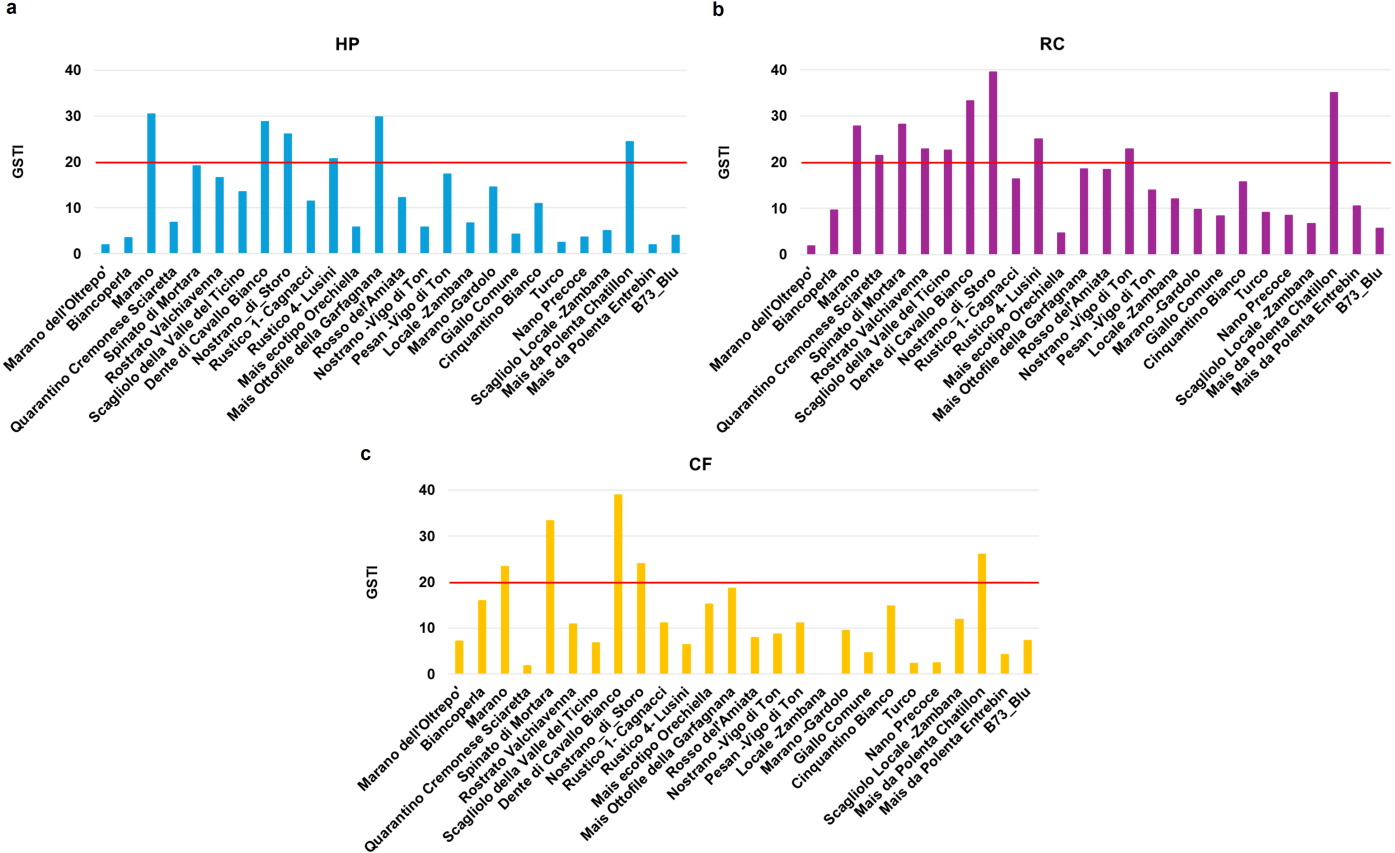
Germination stress tolerance index (GSTI) of *Zea mays* genotypes after seed priming. **(a)** HP, hydropriming. **(b)** RC, priming with red chicory extract. **(c)** CF, priming with cauliflower extract. Data is presented as a ratio of means between the levels of germination under optimal and stress conditions.

Given that GSTI proved a relevant parameter to distinguish between tolerant and susceptible varieties, we used it also to evidence the impact of the seed priming in response to drought (**Figure 6**). The obtained data evidenced that more varieties were able to cross the 20% GSTI threshold after priming. Following HP, three additional genotypes (Mais Ottofile della Garfagnana, Dente di Cavallo Bianco, Rustico 4-Lusini) showed increased tolerance to drought (**Figure 6a**). The RC treatment was the one with the highest number of responsive varieties, reaching to a maximum of seven (Dente di Cavallo Bianco, Spinato di Mortara, Rustico 4-Lusini, Rostrato Valchiavenna, Nostrano -Vigo di Ton, Scagliolo della Valle del Ticino, Quarantino Cremonese Sciaretta) (**Figure 6b**). Differently, the CF priming resulted in only two other genotypes (Dente di Cavallo Bianco, Spinato di Mortara) crossing the 20% threshold (**Figure 6c**).

**Figure 7:**
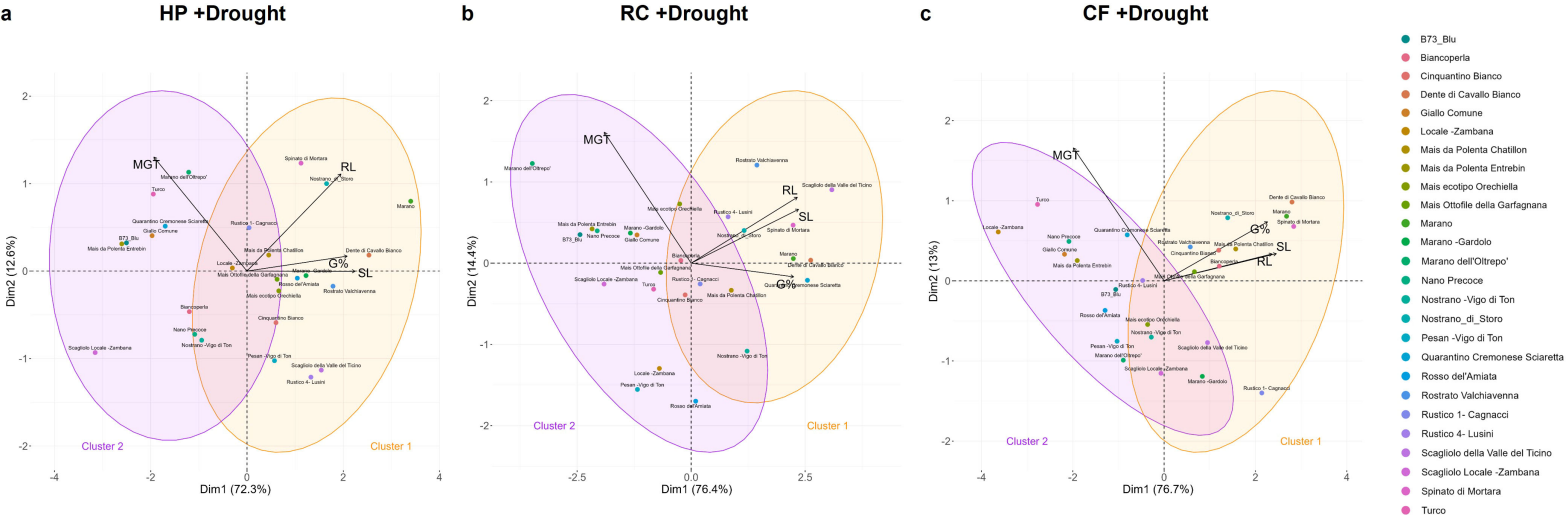
Principal component analysis (PCA) following priming treatments in the presence of drought stress. **(a)** HP, hydropriming. **(b)** RC, priming with red chicory extract. **(c)** CF, priming with cauliflower extract. G%, germination percentage; MGT, mean germination time; SL, shoot length; RL, root length.

Therefore, the presented data indicate a strong genotype-dependent response not only to drought but particularly to the types of tested seed priming protocols. The PCA conducted on the germination data (G%, MGT, SL, RL) reflect the same organization in clusters as previously shown in the absence of stress; however, the number of varieties that perform well under drought stress after priming has increased (**Figure 7**; **Supplementary Table 7**). This highlights the diversity between genotypes, not only from a drought tolerance/sensitivity point of view, but also for their response to specific priming treatments. This is also evidenced in the Venn diagram (**Supplementary Figure 4**) showing the number of genotypes responsive to one or more priming treatments based on their GSTI values, where only one genotype – Dente di Cavallo Bianco – appeared to be responsive to all three priming treatments.

### 3.4 Assessment of molecular markers for seed quality

**Figure 8:**
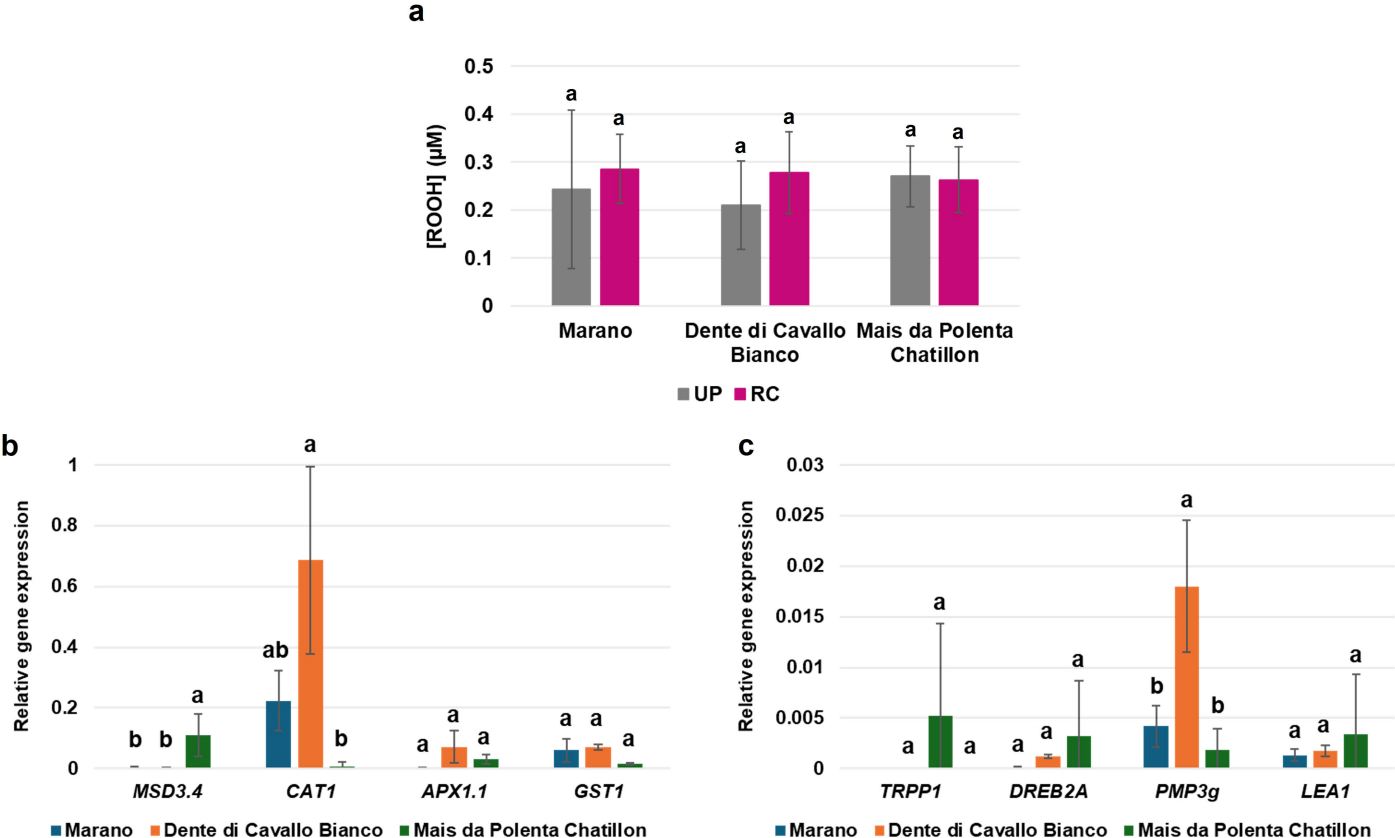
ROS release and gene expression data in dry seeds from Marano, Dente di Cavallo Bianco and Mais da Polenta Chatillon varieties. **(a)** ROS levels in terms of [ROOH] released from seeds. **(b)** Relative expression patterns of genes involved in antioxidant mechanisms. **(c)** Relative expression patterns of genes involved in drought related mechanisms. Data is presented as means ± standard deviations of three independent replicates. Statistical significance (*p* ≤ 0.05) is indicated by the occurrence of different letters, as determined through one-way ANOVA and Tukey’s test. UP, unprimed; RC, priming with red chicory extract; *MSD3.4*, Mn-superoxide dismutase 3.4; *CAT1*, catalase 1; *APX1.1*, ascorbate peroxidase 1.1; *GST1*, glutathione-S-transferase 1; *TRPP1*, trehalose-6-phosphate phosphatase 1; *DREB2A*, Dehydration-responsive element-binding 2A; *PMP3g*, plasma membrane proteolipid 3g; *LEA1*, late embryogenesis abundant protein 1.

The availability of molecular indicators to test seed quality is an emerging field of interest within the seed technology sector (Griffo et al., 2023). Seed quality depends not only on morpho-physiological attributes but also on the inherent ability of seeds to respond to stress during germination and early seedling growth, collectively reflected in the seed vigor trait. Here, we tested some of these indicators, namely the amount of ROS released from seeds and the expression profiles of specific genes, in selected maize varieties (**Figure 8**). The genotypes were selected based on their phenotypic response to drought and priming; specifically, Marano and Mais da Polenta Chatillon are representative for the drought-tolerant category, while Dente di Cavallo Bianco is drought sensitive and responsive to all the priming treatments. Additionally, in terms of germination dynamics, while Marano and Mais da Polenta Chatillon were characterized by fast or medium speed, Dente di Cavallo Bianco is a slow germinating variety.

The FOX-1 assay was used to detect peroxidic radicals released from seeds in the surrounding medium (water). Only unprimed dry seeds and RC-primed seeds were used as this treatment gave better results for multiple varieties. No significant variation between the ROS release neither between genotypes nor for primed/unprimed seeds was observed (**Figure 8a**). Though, a high variability was present as shown by the elevated standard deviation values.

Subsequently, the expression profiles of specific genes involved in the antioxidant response (*MSD3.4, CAT1, APX1.1, GST1*) and drought tolerance (*TRPP1, DREB2A, PMP3g, LEA1*) were targeted. The *MSD3.4* gene, encoding for the manganese superoxide dismutase 3.4 enzyme, is involved in the detoxification of superoxide radicals, protecting cells from oxidative damage (Liu et al., 2021). The *CAT1* (catalase 1) gene product is known to break down H_2_O_2_ into water and oxygen, thus maintaining redox balance (Xie et al., 2024). The *APX1.1* (ascorbate peroxidase 1.1) is responsible for the reaction that reduces H_2_O_2_ using ascorbate (Koussevitzky et al., 2008). The *GST1* gene encodes the glutathione S-transferase 1 enzyme that conjugates glutathione to toxic compounds, aiding in detoxification and stress signaling (Kumar and Trivedi, 2018). Additionally, since the experimental system focused on drought stress responses, genes involved in this physiological process were also selected based on a literature review. The *TRPP1* gene encodes trehalose-6-phosphate phosphatase 1, an enzyme that converts trehalose-6-phosphate to trehalose, thereby know to regulate sugar signaling, energy balance, and stress tolerance (Nuccio et al., 2015). The *DREB2A* gene encodes the Dehydration-Responsive Element-Binding Protein 2A transcription factor, know to activate stress-responsive genes under dehydration (Sakuma et al., 2006). The *PMP3g* (Plasma Membrane Protein 3g) gene was reported to be involved in drought stress tolerance by maintaining membrane potential and ion homeostasis (Lei et al., 2023) while the *LEA1* (Late Embryogenesis Abundant Protein 1) gene function has been related to the stabilization of proteins and membranes during desiccation (Chen et al., 2021). The selection of these genes was based on an *in silico* data mining approach, where the relative expression of candidate genes was recovered from the different compartments of a maize kernel (**Supplementary Figure 5**). The choice to focus on embryo tissue was supported by the observation that the genes of interest showed a consistent expression in the embryo compared to other seed compartments. When the expression of selected genes was measured by qRT-PCR, specific differences were evidenced. In terms of antioxidant related genes, *MSD3.4* showed significantly higher expression in Mais da Polenta Chatillon compared to the other genotypes while *CAT1* was most expressed in Dente di Cavallo Bianco (**Figure 8b**). The *APX1.1* and *GST1* genes did not show any significant differences in expression between the genotypes. Considering the drought tolerance associated genes, only *PMP3g* presented significant differences among varieties, with the highest expression being registered still for Dente di Cavallo Bianco (**Figure 8c**). Even if specific differences can be observed in terms of gene expression among varieties, the information gathered is too limited to draw specific conclusions related to seed quality.

## 4 Discussion

Maize exhibits remarkable biodiversity, reflected in its genetic, physiological, and adaptive variability across cultivars and breeding populations. When considering the Italian maize germplasm, this is considered to be largely derived from traditional landraces and highly differentiated varieties (Mastrangelo et al., 2024). Despite its importance for breeding and genetic studies, this germplasm has been only partially characterized, with most analyses limited to morphological or preliminary genetic investigation traits rather than detailed genetic approaches (Brandolini and Brandolini, 2009; Ardenghi et al., 2018; Bernardi et al., 2018; Stagnati et al., 2021, 2022; Lezzi et al., 2023; Di Pasquale et al., 2024). In view of enhancing biodiversity and agricultural sustainability, landraces are currently experiencing a renewed interest. Characterizing traditional germplasm for stress tolerance can facilitate their reintroduction into local production systems.

In the current study, we evaluated the performance of 26 Italian maize genotypes with different genetic and agronomic characteristics, in terms of germination behavior and drought response. The obtained results evidence that, generally, the seeds exhibited high quality as the majority of varieties were characterized by optimal germination and speed in the absence of stress. A high relevance of the conducted study derived from the identification of germination drought tolerant and susceptible material. Based on a thorough screening process carried out using multiple parameters (G%, MGT, SL, RL, GSTI) and integrative statistical analyses (PCA), we were able to identify four genotypes (Marano, Spinato di Mortara, Nostrano di Storo, and Mais da Polenta Chatillon) with distinct drought tolerance abilities. In the past, genetic analyses using a maize MAGIC population identified a significant variation in germination and seedling traits under drought, emphasizing the role of genetic diversity in determining tolerance and the potential for breeding programs to select superior genotypes (Rida et al., 2021). The importance of the available genetic resources was also highlighted in subtropical maize landraces as reservoirs of novel positive alleles, which, when introduced into breeding populations, can broaden the genetic basis for drought tolerance (Barbosa et al., 2021).

Since the majority of varieties tested presented susceptibility rather than tolerance to drought at germination, another important objective of our work was to develop sustainable priming techniques dedicated to mitigate the effects of soil drought stress. This was carried out with sustainable priming agents based on using plant-based biostimulants obtained from agricultural waste by-products, along with already consolidated methods such as hydropriming (Colombo et al., 2023; Kakar et al., 2023). The plant extracts were obtained with a green extraction technique (Doria et al., 2022) which allowed the resuspension of the bioactive molecules in water rather than ethanol, methanol or other chemical agents. The use of hydrolytic enzymes to disrupt plant cell walls enabled an efficient recovery of valuable metabolites in a sustainable, economical, and scalable process (Domínguez-Rodríguez et al., 2021; Malvis Romero et al., 2023).

Our results highlighted that seed priming had distinct genotype- and treatment-dependent effects under both optimal and drought conditions. Some varieties (e.g., Spinato di Mortara, Rostrato Valchiavenna, Dente di Cavallo Bianco) showed reduced germination percentages under optimal sowing conditions. However, seed priming had generally accelerated germination speed and, in some cases, enhanced seedling growth (e.g., Biancoperla, Rostrato Valchiavenna, Nostrano di Storo). Importantly, these positive effects were much more pronounced under drought conditions in most varieties. The GSTI parameter confirmed that priming enabled more genotypes to surpass the tolerance threshold, with the RC extract showing the overall best effectiveness, followed by HP and CF treatments. The PCA analysis further supported these findings along with providing additional information regarding genotypes clustering in terms of tolerance/susceptibility or responsiveness to priming. Also in this case, the genotype-specific differences were well evident. Dente di Cavallo Bianco was the only variety that responded positively to all three priming methods.

In light of existing research on the use of plant extracts in maize seed treatment, recent studies provide evidence consistent with the results obtained in our study. For instance, *Plantago ovata* leaf extract was shown to improve germination traits, seedling growth, and physio-biochemical performance under water stress (Nawaz et al., 2021). Similarly, *Moringa oleifera* extracts were able to mitigated drought-induced damage by boosting physiological, biochemical, and antioxidant activities (Bhowmick et al., 2024). Recently, there has been growing interest in repurposing fruit and vegetable waste by-products for applications in the seed industry, either for the production of nanoparticles (Yadav and Mathur, 2024) or for their direct use as seed priming agents (Wazeer et al., 2024). The novelty of our work lies in the sustainable and cost-effective extraction of bioactive compounds from specific agricultural waste products, namely, red chicory and cauliflower, tested for the first time as seed priming agents, specifically on a high number of maize varieties. The different effects on germination can be attributed to both the genetic background of the investigated varieties as well as to the chemical composition of the extracts. The RC and CF extracts contain high levels of polyphenols, including quercetin, rutin, chlorogenic acid, and cinnamic acid, among others. Such molecules are known to protect plants by reducing oxidative stress. Priming *Medicago truncatula* seeds with quercetin or rutin was shown to enhance their antioxidant profiles and improve seed storability (Gaonkar et al., 2024). In maize, chlorogenic acid was found to mitigate arsenic- and heavy metal-induced damage by enhancing enzyme activity, and maintaining the redox balance (Kısa et al., 2016; Arikan et al., 2022; Elbasan et al., 2024). Similarly, cinnamic acid mitigated oxidative stress by enhancing antioxidant enzymes, reducing lipid peroxidation, and stimulating salicylic and ascorbic acid pathways to boost radical scavenging and stress tolerance (Singh et al., 2013; Araniti et al., 2018).

The last part of our study focused on assessing putative markers of seed quality in selected varieties with contrasting profiles. This is relevant for understanding the molecular bases of seed vigor and performance. Identifying reliable markers could facilitate the selection and breeding of superior genotypes, improve seed quality assessment protocols, and potentially better understand the response to priming (Griffo et al., 2023). To this end, Marano and Mais da Polenta Chatillon were selected as drought-tolerant genotypes with fast or medium germination speed, whereas Dente di Cavallo Bianco was selected as a drought-sensitive genotype with slow germination and high responsiveness to all priming treatments. Marano is a variety developed in the late 20^th^ century in Marano Vicentino to be productive in rainfed cultivation on soils characterized by high permeability (Bertolini, 2002). Mais da Polenta Chatillon was sampled in Valle d’Aosta (Lezzi et al., 2023) but precise information regarding its selection and historical cultivation are not available. Both landraces are characterized by small flint seeds with orange or dark-red color and, according to morphology, they can be ascribed to the Microsperma racial complex (Brandolini and Brandolini, 2009). Marano and Chatillon, as many other landraces, have a historical use for human consumption for the production of polenta (Lezzi et al., 2023). Dente di Cavallo Bianco originated in Friuli Venezia Giulia as a forage maize landrace, though it is also used for human consumption (Ardenghi et al., 2018). This variety is characterized by tall plants, often reaching three meters in height, kernels are of dent-like type with white or red pigmentation; it is characterized by a beak on the kernel tip, which classifies it within the Rostrata group (Ardenghi et al., 2018). Historically, cultivation of landraces belonging to this group was carried out only in areas with high water availability to sustain their long cycle and vigor; this may explain the relative drought susceptibility observed in this study.

When the levels of ROS released from the seeds of these genotypes were measured, no significant differences were observed for both primed/unprimed seeds. However, the data showed high variability, that may reflect elevated seed-to-seed heterogeneity, as reported in previous studies (Griffo et al., 2023). Finally, genes encoding for proteins involved in the antioxidant response were investigated. We specifically focused on analyzing the expression of these genes only in dry seeds because we were aiming to look into molecular markers that could serve as molecular indicators of seed quality. Our analyses showed that *MSD3.4* was highly expressed in Mais da Polenta Chatillon, while *CAT1* expression more expressed in Dente di Cavallo Bianco. Among drought-related genes, only *PMP3g* varied significantly among varieties, presenting the highest expression in Dente di Cavallo Bianco. These findings may suggest that elevated expression of *CAT1* and *PMP3g* could be potentially used to distinguish between tolerant/susceptible seeds, thus high vs. low vigor; however, further studies are necessary to verify this hypothesis. Previous studies in maize shown that *PMP3g* is involved in drought tolerance mechanisms by regulating ABA-GA homeostasis, promoting root elongation, and activating both ABA-dependent and independent stress-response pathways (Lei et al., 2023). Additionally, it can also influence genes involved in ROS scavenging, particularly *MSD3.4* and *CAT1*, which contributing to drought tolerance by detoxifying ROS via the salicylic acid pathway (Liu et al., 2021; Li et al., 2024).

In conclusion, this study evidenced that the tested Italian maize variety were characterized by a general high seed quality in terms of germination performance in the absence of stress. However, most of them appeared to be susceptible to drought stress applied as low level of soil moisture content during germination. Importantly, Marano, Mais da Polenta Chatillon, Nostrano di Storo, and Spinato di Mortara were identified as varieties with a relevant potential for drought tolerance at this key developmental stage. Another relevant finding of our study is the fact that seed priming with the RC and CF extracts was able to mitigate the effects of drought in a genotype- and treatment-specific manner. This may be due to both the varietal genetic background and the composition of the extracts, as well as the interaction between the two factors. To advance sustainable biostimulant strategies within a circular bioeconomy framework, future research should be conducted to correlate specific metabolites present in these extracts with physiological responses, extend molecular profiling to stress-related pathways, and validate the effectiveness of the treatments under field conditions.

## Data Availability

The raw data supporting the conclusions of this article will be made available by the authors, without undue reservation.
