# Supplementary material for: Seed priming with plant waste extracts enhances maize drought tolerance in a genotype-specific manner
Source: Front Plant Sci. 2025 Nov 10;16:1717255. doi: 10.3389/fpls.2025.1717255 (PMC12640863; doi:10.3389/fpls.2025.1717255)
Supplement: Supplementary file 1 [file DataSheet1.docx]

**Seed priming with plant waste extracts enhances maize drought tolerance in a genotype-specific manner**

Hisham Wazeer^1^, Ahmad Zeidan^1^, Jacopo Allevi^1^, Andrea Pagano^1^, Conrado Dueñas Jr.^1^, Adriano Marocco^2,3^, Lorenzo Stagnati^2,3^, Enrico Doria^1^, Anca Macovei^1^*

^1^ Department of Biology and Biotechnology ‘L. Spallanzani’, University of Pavia, via Ferrata 9, 27100, Pavia, Italy

^2^ Department of Sustainable Crop Production, Università Cattolica del Sacro Cuore, via Emilia Parmense 84, 29122, Piacenza, Italy

^3^ Research Centre for Biodiversity and Ancient DNA, Università Cattolica del Sacro Cuore, Via Emilia Parmense 84, 29122 Piacenza, Italy

*** Correspondence:**Anca Macovei
anca.macovei@unipv.it

Supplementary Material

**Supplementary Table 1.** Agronomic and geographical characteristics of the studied maize genotypes. Landrace’s sources are reported as: CREA-CI (Centro di Ricerca per la Cerealicoltura e Colture Industriali - Bergamo), UCSC (Department of Sustainable Crop Production -Università Cattolica del Sacro Cuore, UNIPV (Banca del Germoplasma Vegetale, Università degli Studi di Pavia), (Institut Agricole Régional, Aosta).

| **Code** | **Name** | **Kernel type** | **Seed color** | **Region** | **Collector** | **Reference** |
| --- | --- | --- | --- | --- | --- | --- |
| 1915 | Marano dell'Oltrepo' | Flint | Yellow | Lombardy | UNIPV | - |
| Biancoperla | Biancoperla | Flint | White | Veneto | UCSC | - |
| Marano | Marano | Semi-flint | Orange to red | Veneto | UCSC | - |
| Q_C | Quarantino Cremonese Sciaretta | Flint | Yellow | Lombardy | UCSC | - |
| R16 | Spinato di Mortara | Flint | Yellow | Lombardy | UNIPV | Ardenghi et al., 2018; Stagnati et al., 2021 |
| R17 | Rostrato Valchiavenna | Flint | Red to dark orange | Lombardy | UNIPV | Ardenghi et al., 2018; Stagnati et al., 2021 |
| R2 | Scagliolo della Valle del Ticino | Flint | Yellow | Lombardy | UNIPV | Ardenghi et al., 2018 |
| R3 | Dente di Cavallo Bianco | Dent | White | North Italy | UNIPV | Ardenghi et al., 2018; Stagnati et al., 2021 |
| Va1214 | Nostrano_di_Storo | Flint | Orange | Trentino-Alto Adige | CREA-CI | Brandolini & Brandolin, 2009 |
| Va1281 | Rustico 1- Cagnacci | Flint | Yellow | Tuscany | UCSC | Brandolini & Brandolin, 2009 |
| Va1284 | Rustico 4- Lusini | Flint | Yellow | Tuscany | UCSC | Brandolini & Brandolin, 2009 |
| Va1290 | Mais ecotipo Orechiella | Flint | Yellow | Tuscany | UCSC | Brandolini & Brandolin, 2009 |
| Va1291 | Mais Ottofile della Garfagnana | Flint | Yellow | Tuscany | UCSC | Brandolini & Brandolin, 2009 |
| Va1292 | Rosso del'Amiata | Flint | Red | Tuscany | UCSC | Brandolini & Brandolin, 2009 |
| Va145 | Nostrano -Vigo di Ton | Flint | Yellow | Trentino-Alto Adige | CREA-CI | Brandolini & Brandolin, 2009 |
| Va148 | Pesan -Vigo di Ton | Flint | Yellow | Trentino-Alto Adige | CREA-CI | Brandolini & Brandolin, 2009 |
| Va153 | Locale -Zambana | Flint | Yellow | Trentino-Alto Adige | CREA-CI | Brandolini & Brandolin, 2009 |
| Va154 | Marano -Gardolo | Flint | Orange | Trentino-Alto Adige | CREA-CI | Brandolini & Brandolin, 2009 |
| Va216 | Giallo Comune | Flint | Yellow | North Italy | CREA-CI | Brandolini & Brandolin, 2009; Stagnati et al., 2022b |
| Va220w | Cinquantino Bianco | Flint | White | North Italy | CREA-CI | Brandolini & Brandolin, 2009 |
| Va221 | Turco | Flint | Yellow | North Italy | CREA-CI | Brandolini & Brandolin, 2009 |
| Va225 | Nano Precoce | Flint | Yellow | North Italy | CREA-CI | Brandolini & Brandolin, 2009 |
| Va555 | Scagliolo Locale -Zambana | Flint | Yellow | Trentino-Alto Adige | CREA-CI | Brandolini & Brandolin, 2009 |
| VDA_3 | Mais da Polenta Chatillon | Flint | Yellow | Aosta Valley | IAR | Lezzi et al., 2023 |
| VDA_4 | Mais da Polenta Entrebin | Flint | Yellow | Aosta Valley | IAR | Lezzi et al., 2023 |
| B73_Blu | B73_Blu | Dent | Gray-blue | USA (adapted in Italy) | UCSC | Bernardi et al., 2018 |

**Supplementary Table 2.** Gradient conditions of the mobile phases consisted in 5% acetic acid (A) and pure methanol (B) used for the HPLC analyses.

| **Time (Min)** | **A (%)** | **B (%)** |
| --- | --- | --- |
| 1 | 90 | 10 |
| 5 | 90 | 10 |
| 7 | 80 | 20 |
| 8 | 80 | 20 |
| 10 | 75 | 25 |
| 15 | 70 | 30 |
| 20 | 20 | 80 |
| 25 | 50 | 50 |
| 28 | 70 | 30 |
| 30 | 90 | 10 |

**Supplementary Table 3.** Germination parameters with their corresponding significance and equations. ni, number of germinated seeds at a specific timepoint; ti, a specific timepoint (in days).

| **Parameter** | **Significance** | **Equation** |
| --- | --- | --- |
| G% | Germination percentage | (number of germinated seeds/total number of seeds) x 100 |
| SL | Shoot length | (measured by ImageJ software) |
| RL | Root length | (measured by ImageJ software) |
| MGT | Mean germination time | ∑ni/∑(niti) |
| GSTI | Germination stress tolerance index | (average number of germinated seeds in normal condition/average number of germinated seeds in stress condition) x100 |

**Supplementary Table 4.** List of oligonucleotide sequences used for the qRT-PCR reactions.

| **Gene** | **Accession No.** | **Primer forward (5’-3’)** | **Primer reverse (3’-5’)** |
| --- | --- | --- | --- |
| *MSD3.4* | Zm00001d009990 | ATCCTGTATTCCTGCTTGCG | AAATGGTTCTTTTGGGCATCAC |
| *CAT1* | Zm00001d014848 | CACCCAGAGAGCCTACACAT | AGCAAGCATTTCACACCACA |
| *APX1.1* | Zm00001d028709 | CTGGGGTTTGCTGATGCTTA | ACGAGAAAGACAATACGACAATCT |
| *GST1* | Zm00001d012675 | AACCACCGACCAGAAAGTTG | ACAGGCAGAGAGTGACAGAC |
| *TRPP1* | Zm00001d032298 | CCACGGATGTTCCTTCTCCT | GCTATGGGCTCTGGCTC |
| *DREB2A* | Zm00001d008665 | GCGTCAGAGGTGGGAGAG | GGTGGCGGTGGTATCCTA |
| *PMP3g* | Zm00001d024778 | CATCATCTACGCCATCTACGC | GCAAAACAGGAACACGACTGA |
| *LEA1* | Zm00001d027740 | CCACACTCACCATCAAGTCTC | CTCCTGGCTCATTCTCGCT |
| *18s* | LOC118472325 | CGTCGCTCCTACCGATTGA | CACCTACGGAAACCTTGTTACG |

**Supplementary Table 5**. PCA loading tables explaining the dimensions (Dim) of each germination parameters in the absence of priming and presence/absence of drought. G%, germination percentage; SL, shoot length; RL, root length; MGT, mean germination time.

| Feature | Dim.1 | Dim.2 | Dim.3 | Dim.4 |
| --- | --- | --- | --- | --- |
| **-Drought** | | | | |
| G% | 0.7101 | -0.6490 | 0.2338 | 0.1403 |
| MGT | -0.8764 | 0.1227 | 0.3175 | 0.3404 |
| SL | 0.85487 | 0.2843 | -0.2872 | 0.3253 |
| RL | 0.7737 | 0.4206 | 0.4624 | -0.1026 |
| **+Drought** | | | | |
| G% | 0.9043 | -0.0796 | 0.2988 | 0.2190 |
| MGT | -0.6826 | 0.6850 | 0.1355 | 0.0883 |
| SL | 0.8742 | 0.3001 | 0.0945 | -0.3133 |
| RL | 0.8610 | 0.3220 | -0.3024 | 0.1581 |

**Supplementary Table 6**. Multifactorial ANOVA on germination data presenting the impact of each factor and the combination of factors. Df, degrees of freedom; Sum Sq, sum of squares; Mean Sq, mean of squares; Partial eta^2, partial eta squared; Pr(>F), *p*-value.

|  | **Df** | **Sum Sq** | **Mean Sq** | **F value** | **Partial eta****^2** | **Pr(>F)** |  |
| --- | --- | --- | --- | --- | --- | --- | --- |
| **Germination percentage (G%)** | | | | | | | |
| Genotype | 25 | 21157.41 | 846.2965 | 10.69006 | 0.391146615 | 1.58E-31 | *** |
| Treatment | 3 | 1363.582 | 454.5272 | 5.741397 | 0.039758145 | 0.000742 | *** |
| Condition | 1 | 829062.5 | 829062.5 | 10472.37 | 0.961794094 | 4.8E-297 | *** |
| Genotype:Treatment | 75 | 7347.877 | 97.97169 | 1.237537 | 0.182414496 | 0.101977 |  |
| Genotype:Condition | 25 | 14455.17 | 578.2067 | 7.303664 | 0.305035352 | 8.4E-21 | *** |
| Treatment:Condition | 3 | 5267.748 | 1755.916 | 22.17999 | 0.137895268 | 2.43E-13 | *** |
| Genotype:Treatment:Condition | 75 | 6718.71 | 89.5828 | 1.131572 | 0.169441708 | 0.227587 |  |
| Residuals | 416 | 32933.33 | 79.16667 |  |  |  |  |
| **Mean germination time (MGT)** | | | | | | | |
| Genotype | 25 | 535.0743 | 21.40297 | 5.138899 | 0.235957706 | 1.69E-13 | *** |
| Treatment | 3 | 57.27697 | 19.09232 | 4.584107 | 0.032000574 | 0.003593 | ** |
| Condition | 1 | 583.5476 | 583.5476 | 140.111 | 0.251947925 | 4.64E-28 | *** |
| Genotype:Treatment | 75 | 308.5071 | 4.113428 | 0.987643 | 0.151147225 | 0.511478 |  |
| Genotype:Condition | 25 | 269.7258 | 10.78903 | 2.59047 | 0.134706526 | 5.83E-05 | *** |
| Treatment:Condition | 3 | 40.46271 | 13.48757 | 3.238394 | 0.02282085 | 0.022139 | * |
| Genotype:Treatment:Condition | 75 | 357.882 | 4.77176 | 1.14571 | 0.171196239 | 0.206448 |  |
| Residuals | 416 | 1732.596 | 4.164895 |  |  |  |  |
| **Shoot length (SL)** | | | | | | | |
| Genotype | 25 | 14479.5 | 579.1798 | 140.9347 | 0.894399315 | 1.1E-185 | *** |
| Treatment | 3 | 146.3286 | 48.7762 | 11.86896 | 0.078844841 | 1.79E-07 | *** |
| Condition | 1 | 28396.91 | 28396.91 | 6909.962 | 0.943215647 | 3.1E-261 | *** |
| Genotype:Treatment | 75 | 2332.7 | 31.10266 | 7.568367 | 0.577075694 | 1.64E-43 | *** |
| Genotype:Condition | 25 | 1694.814 | 67.79254 | 16.4963 | 0.497831674 | 9.69E-48 | *** |
| Treatment:Condition | 3 | 252.6581 | 84.21938 | 20.49352 | 0.12876036 | 2.1E-12 | *** |
| Genotype:Treatment:Condition | 75 | 2955.986 | 39.41315 | 9.5906 | 0.633575414 | 5.34E-55 | *** |
| Residuals | 416 | 1709.577 | 4.109561 |  |  |  |  |
| **Root length (RL)** | | | | | | | |
| Genotype | 25 | 7962.02 | 318.4808 | 50.87761 | 0.753545763 | 5.6E-110 | *** |
| Treatment | 3 | 52.84838 | 17.61613 | 2.814193 | 0.01989098 | 0.038987 | * |
| Condition | 1 | 47187.37 | 47187.37 | 7538.227 | 0.947700761 | 1.1E-268 | *** |
| Genotype:Treatment | 75 | 3083.6 | 41.11467 | 6.568107 | 0.542156823 | 2.52E-37 | *** |
| Genotype:Condition | 25 | 2102.227 | 84.08906 | 13.43331 | 0.446685387 | 1.63E-39 | *** |
| Treatment:Condition | 3 | 348.7426 | 116.2475 | 18.57066 | 0.118105898 | 2.53E-11 | *** |
| Genotype:Treatment:Condition | 75 | 2727.618 | 36.36825 | 5.809861 | 0.511587819 | 2.13E-32 | *** |
| Residuals | 416 | 2604.054 | 6.259744 |  |  |  |  |

**Supplementary Table 7**. PCA loading tables explaining the dimensions (Dim) of each germination parameters under drought after priming. G%, germination percentage; SL, shoot length; RL, root length; MGT, mean germination time.

| Feature | Dim.1 | Dim.2 | Dim.3 | Dim.4 |
| --- | --- | --- | --- | --- |
| **Hydropriming (HP)** | | | | |
| G% | 0.84345154 | 0.06923343 | 0.45365999 | -0.19881474 |
| MGT | -0.78338375 | 0.52812919 | 0.24105851 | 0.10401303 |
| SL | 0.91009345 | -0.00083213 | 0.09376292 | 0.35281185 |
| RL | 0.79154887 | 0.44986496 | -0.35264016 | -0.09085862 |
| **Priming with red chicory extract (RC)** | | | | |
| G% | 0.8689592 | -0.06516122 | 0.44948694 | 0.01280163 |
| MGT | -0.73651026 | 0.62257043 | 0.17644594 | 0.01907857 |
| SL | 0.91067176 | 0.25594869 | -0.16662817 | 0.19733406 |
| RL | 0.90006503 | 0.31338481 | -0.12097792 | -0.19640702 |
| **Priming with cauliflower extract (CF)** | | | | |
| G% | 0.87149097 | 0.28378297 | -0.38840692 | -0.09545041 |
| MGT | -0.76479538 | 0.6384444 | 0.08364473 | 0.02191635 |
| SL | 0.94588678 | 0.13047146 | 0.0899787 | 0.28315939 |
| RL | 0.90969002 | 0.12922402 | 0.34886011 | -0.1845585 |


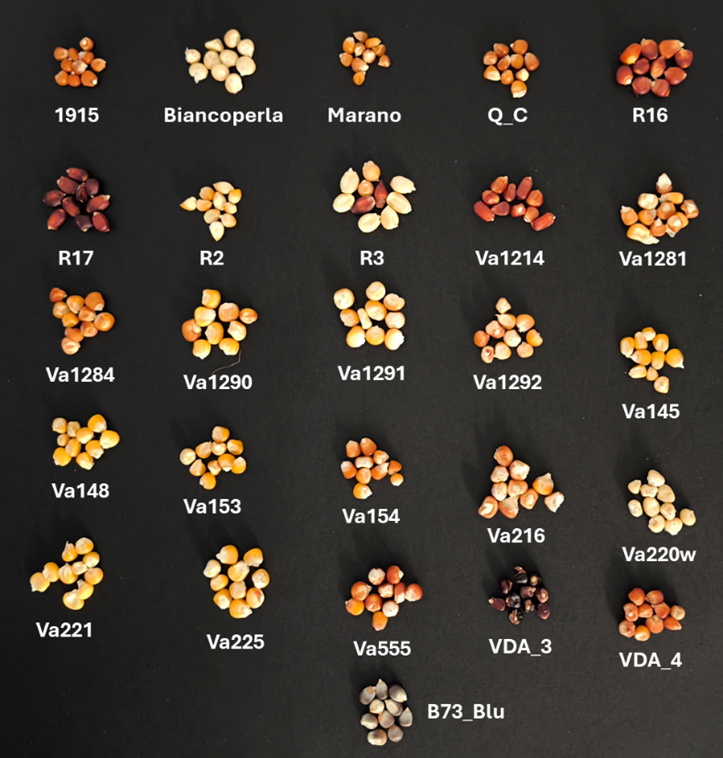


**Supplementary Figure 1.** Representative image of the 26 maize genotypes. The codes included in the image are same as the varietal code from Supplementary Table 1.


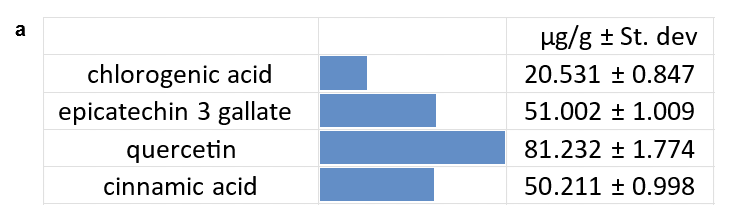


Total polyphenols (mg/g) = 2.201 ± 0.058


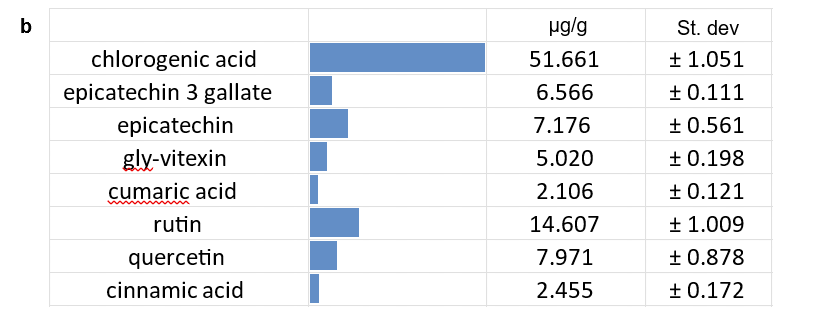


Total polyphenols (mg/g) = 1.988 ± 0.017

**Supplementary Figure 2.** Polyphenol composition of the plant waste extracts as revealed by the HPLC analysis. **(a)** Red chicory (RC) extract. **(b)** Cauliflower (CF) extract. The total polyphenol content is also indicted.


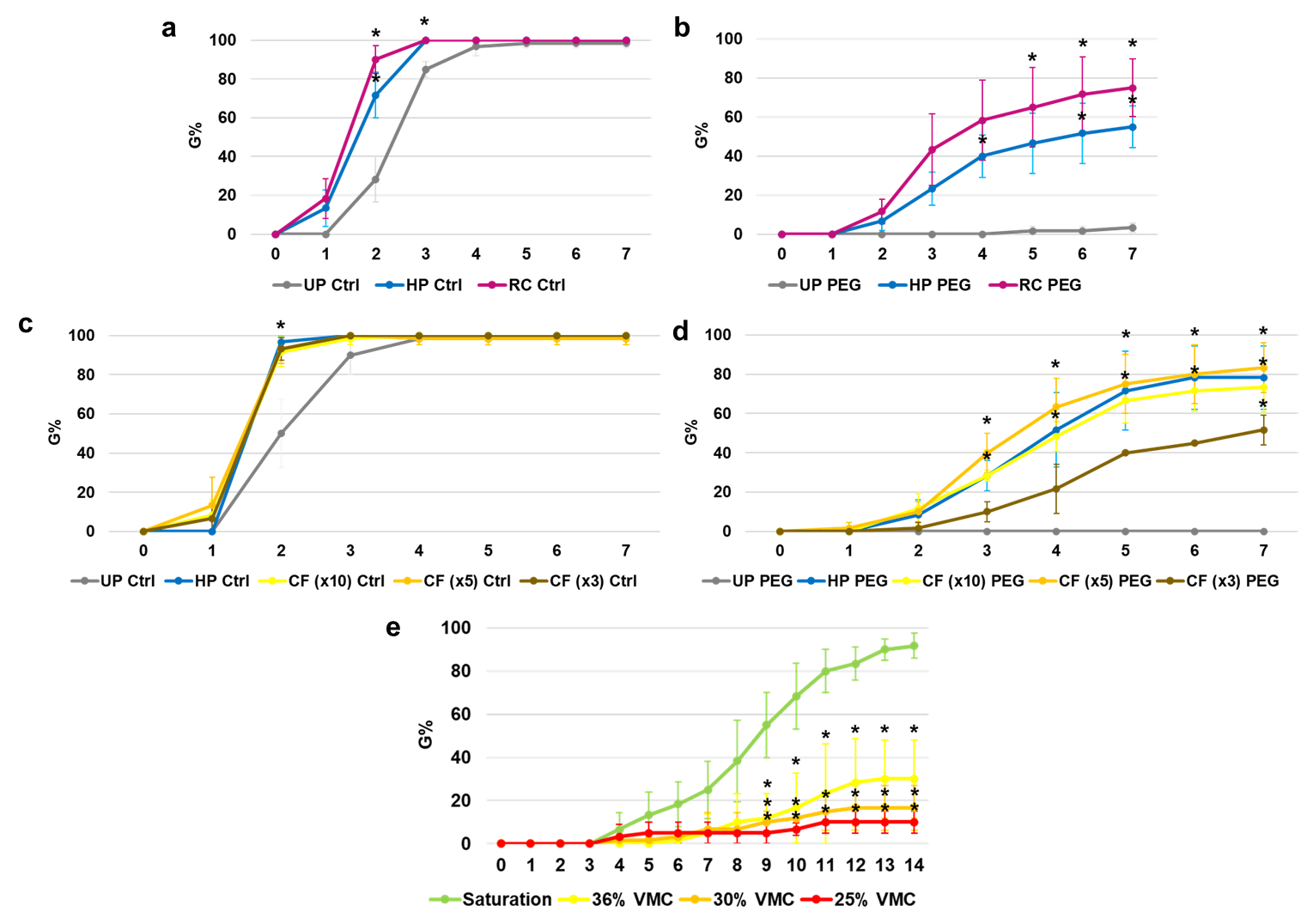


**Supplementary Figure 3.** Selection of plant extract dilutions and drought stress level based on germination percentage (G%). Unprimed (UP) and hydroprimed (HP) seed germination is also presented comparatively. **(a)** Red chicory (RC) extract, diluted 6x based on previous data on other species (data not shown), used under optimal conditions. **(b)** RC extract priming under stress conditions imposed by using 18% polyethylene glycol (PEG). **(c)** Cauliflower (CF) extract at dilution 10x, 5x and 3x; seeds were germinated under optimal conditions. **(d)** CF extract priming in the presence of PEG. **(e)** Selection of drought stress level based on VMC of soil. Data are represented as means ± SD of three replicates. Statistical significance, as per the Student’s *t*-Test, is shown with asterisk (*, *p* ≤ 0.05) where the UP treatment is used as control. UP, unprimed seeds; HP, hydropriming; RC, red chicory extract; CF, cauliflower extract; Ctrl, optimal conditions; PEG, polyethylene glycol; VMC, volumetric moisture content.

**Supplementary Figure 4.** Venn diagram representing the number of varieties grouped based on the different responsiveness to seed priming according to GSTI (germination stress tolerance index) values. HP, hydropriming; RC, red chicory extract; CF, cauliflower extract.


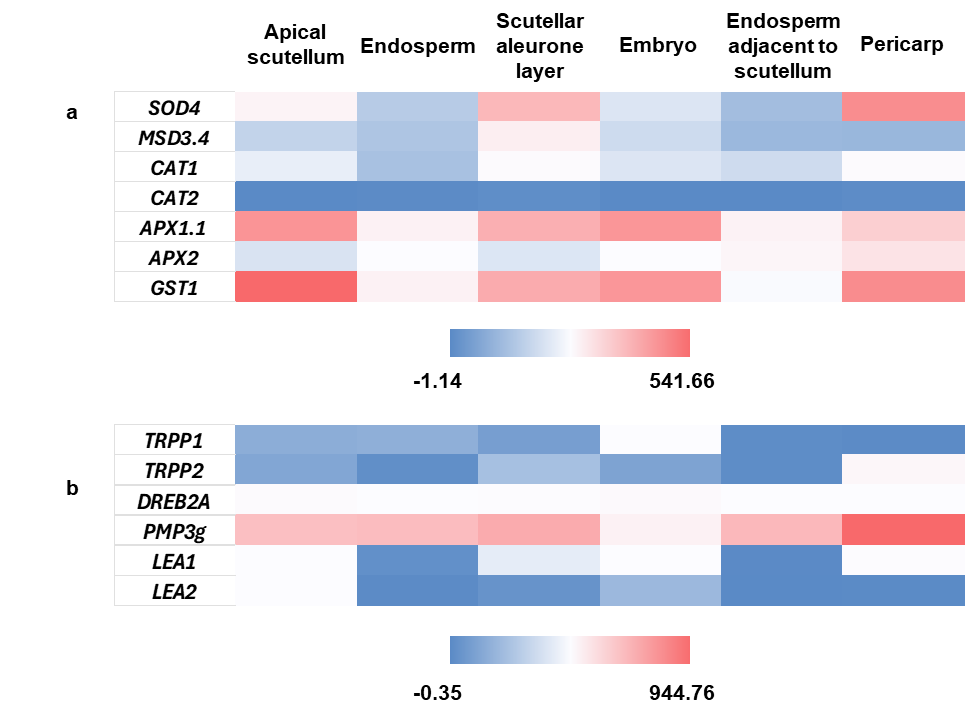


**Supplementary Figure 5.** Gene expression data retrieved from publicly available transcriptomics datasets (Maize eFP Browser, <https://bar.utoronto.ca/efp_maize/cgi-bin/efpWeb.cgi?dataSource=Maize_Kernel>). Genes involved in antioxidant **(a)** and drought-stress **(b)** related mechanisms were targeted for this data mining. *SOD4*, superoxide dismutase isoform 4; *MSD3.4*, Mn-superoxide dismutase isoform 3.4 (NCBI accession: Zm00001d009990); *CAT1*, catalase isoform 1 (NCBI accession: Zm00001d014848); *CAT2*, catalase isoform 2 (NCBI accession: Zm00001d027511); *APX1.1*, ascorbate peroxidase isoform 1.1 (NCBI accession: Zm00001d028709); *APX2*, ascorbate peroxidase isoform 2 (NCBI accession: Zm00001d007234); *GST1*, glutathione-S-transferase isoform 1 (NCBI accession: Zm00001d012675); *TRPP1*, trehalose-6-phosphate phosphatase isoform 1 (NCBI accession: Zm00001d032298); *TRPP2*, trehalose-6-phosphate phosphatase isoform 2 (NCBI accession: Zm00001d027355); *DREB2A*, dehydration-responsive element-binding 2A (NCBI accession: Zm00001d008665); *PMP3g*, plasma membrane proteolipid 3g (NCBI accession: Zm00001d024778); *LEA1*, late embryogenesis abundant isoform 1 (NCBI accession: Zm00001d027740); *LEA2*, late embryogenesis abundant isoform 2 (NCBI accession: Zm00001d027832).
